# Pairing Behavior of the Monogamous King Quail, *Coturnix chinensis*

**DOI:** 10.1371/journal.pone.0155877

**Published:** 2016-06-03

**Authors:** Elizabeth Adkins-Regan

**Affiliations:** Department of Psychology and Department of Neurobiology and Behavior, Cornell University, Ithaca, New York, United States of America; The University of Texas at Austin, UNITED STATES

## Abstract

Animals with socially monogamous mating systems are valuable for discovering proximate mechanisms of prosocial behavior and close social relationships. Especially powerful are comparisons between related species that differ in monogamous tendency. Birds are the most socially monogamous vertebrates. Thus far most research on mechanisms of pairing has used zebra finches, which do not have a relative with a different mating system, however. The goal of the experiments reported here was to develop a new comparative avian system by studying the pairing behavior of a reportedly strongly monogamous quail, the king quail (*Coturnix chinensis*), a species in the same clade as the less monogamous Japanese quail (*Coturnix japonica*), the subject of much prior research. In Experiment 1 male-female pairs of king quail housed together were initially avoidant or aggressive but most rapidly progressed to allopreening and huddling. A separation-reunion paradigm reliably elicited both of these behaviors in males that had cohabited for one week. In Experiment 2 the allopreening and huddling behavior of males in cohabiting pairs was highly selective, and a majority of the males were aggressive toward a familiar female that was not the cohabitation partner. In Experiment 3 males were separated from their female cohabitation partners for 9–10 weeks and then given two-choice tests. All but one male spent more time near an unfamiliar female, which may have reflected aggression and shows recognition of and memory for the past pairing experience. Thus king quail show robust, selective and easy to measure pairing behavior that can be reliably elicited with simple separation-reunion testing procedures. Copulation is rarely seen during tests. The behavior of king quail is a striking contrast to that of Japanese quail, providing a new comparative system for discovering mechanisms of behavior related to close social relationships and monogamy.

## Introduction

There is currently a great deal of interest in diverse forms of non-aggressive social behavior. Positive or prosocial behavior between unrelated individuals raises important questions about both ultimate and proximate causation. One major goal of research on proximate causation has been to discover the neuroendocrine and other neural mechanisms of such behavior. Important advances have been made through experiments focusing on male-female pair relationships in socially monogamous species such as prairie voles (*Microtus ochrogaster*) and California mice (*Peromyscus californicus*) [1–3]. Some of the power of these research programs has come from comparing these animals with related species that have non-monogamous mating systems and lack close social relationships between the sexes [4–6].

Birds are the most socially monogamous vertebrates. Over 90% of the species have some form of this mating system, and birds have been prominent in research on the ecology and evolution of monogamy [7–9]. Until recently, however, very little was known about any of the hormonal or neural mechanisms of avian pairing. Progress is beginning through research with zebra finches [10–13]. Zebra finches are good subjects because they form permanent pairs marked by distinctive contact behavior such as allopreening and clumping (huddling together on a perch). One drawback, however, is the absence of a contrast with a related species. All the species in the zebra finch family (Estrildidae) appear to have a similar socially monogamous mating system [14,15].

Zebra finches are also one of the two species used for a majority of the avian research on neuroendocrine mechanisms of behavior more generally. The other is the Japanese quail (*Coturnix japonica*). Male Japanese quail interacting with females are vigorous rapid copulators but non-copulatory social contact behavior like allopreening and huddling has not been reported [16,17]. The limited information available from the field suggests that the mating system of wild birds is probably flexible, with short-term serial pairings at best [17,18]. In contrast, king quail (also called Asian blue quail, blue-breasted quail, or Chinese painted quail) are invariably described as monogamous with strong pair bonds, based on field sightings and observations of zoo-housed animals [18,19]. Like Japanese quail, king quail are well suited to laboratory research [20]. Although an ethogram was published some years ago [21], there has been little subsequent behavioral research published on this species and none on its social behavior.

The overall goal of the experiments reported here was to develop king quail as subjects for the study of avian pairing behavior, allowing a comparison with Japanese quail. The specific goals were: (1) to develop suitable testing paradigms for eliciting and measuring the behavior; (2) to determine whether king quail form selective male-female social relationships; and (3) to determine whether paired birds remember the former partner after an extended separation. The focus was on the behavior of males because most of the extensive body of work on neuroendocrine mechanisms of behavior of Japanese quail concerns males.

## General Methods

### Animals

Fertilized eggs were obtained from Stromberg’s Chicks and Game Birds (Pine River, MN, USA) and incubated at 37.5°C and approximately 30% relative humidity. Chicks were housed in heated brooders until 4 weeks of age and then housed individually in wire cages (57 cm long X 32 cm deep X 40 cm high) on a 14h:10h light:dark cycle throughout the experiments. Opaque barriers were placed between adjacent cages so that each bird could not see any other birds at close range. The birds were of mixed plumage types: wildtype, pale wildtype (all those were males), speckled or pinto (all those were females), silver, white or tuxedo. Wildtype plumage is distinctly sexually dimorphic; silver, white and tuxedo are monomorphic. An effort was made to match plumages to control for plumage preferences, which have been shown in Japanese quail, but such matching was not always possible [22]. The experiments began once birds were sexually mature, as indicated by egg laying by females.

### Ethics statement

All animal housing and procedures followed state and federal guidelines and were approved by the Institutional Animal Care and Use Committee of Cornell University (protocol number: 2012–0098).

### Testing procedures

All tests were conducted between 1300 and 1800 h in an otherwise empty room adjacent to the main housing room. King quail are very vocal, and housing room vocalizations were audible in the testing room. To move a bird from one cage to another for testing, care was taken to avoid the disturbance that handling might produce by placing the two cages together with the doors open and letting the bird move to the other cage. All tests were video recorded with no human in the room.

### Behavioral Measurement

The behaviors were defined and coded from the video recordings as follows:

#### Loud call

A loud musical one-to-three-syllable vocalization. This is probably what is called “crowing” or “advertisement call” in some sources [19]. When loud calling the bird is in a vertical posture with outstretched neck. Each call was counted.

#### Growl

A softer and lower pitched vocalization performed only by males and said to be a separation call [19]. When growling the male is in a crouched posture with a puffed throat. Each growl was counted.

#### Wings-down display

A male-specific display in which both wings are fanned out and the body feathers are fluffed up as the male approaches the target bird. It resembles in form the strutting display of other galliform birds. It is termed “wings-down attack” by Schleidt et al. [21], implying that it occurs in aggressive contexts. Each display was counted.

#### Aggression

Threat postures or repeated lunging at, chasing or attacking another bird. The presence or absence of such behavior was noted.

#### Allopreening

One bird preens another’s feathers with its beak, usually but not always the feathers of the head or neck (see S1 Video). Once initiated by one bird, it can become mutual. Each bout of allopreening initiated was counted, with bouts defined as separated by at least 10 sec. Allopreening can occur with or without huddling.

#### Huddling

Two birds squat in a resting position side by side with their bodies in contact (see S1 Video). Total duration in sec was recorded. Huddling can occur with or without allopreening.

#### Proximity time

**Fig 1:**
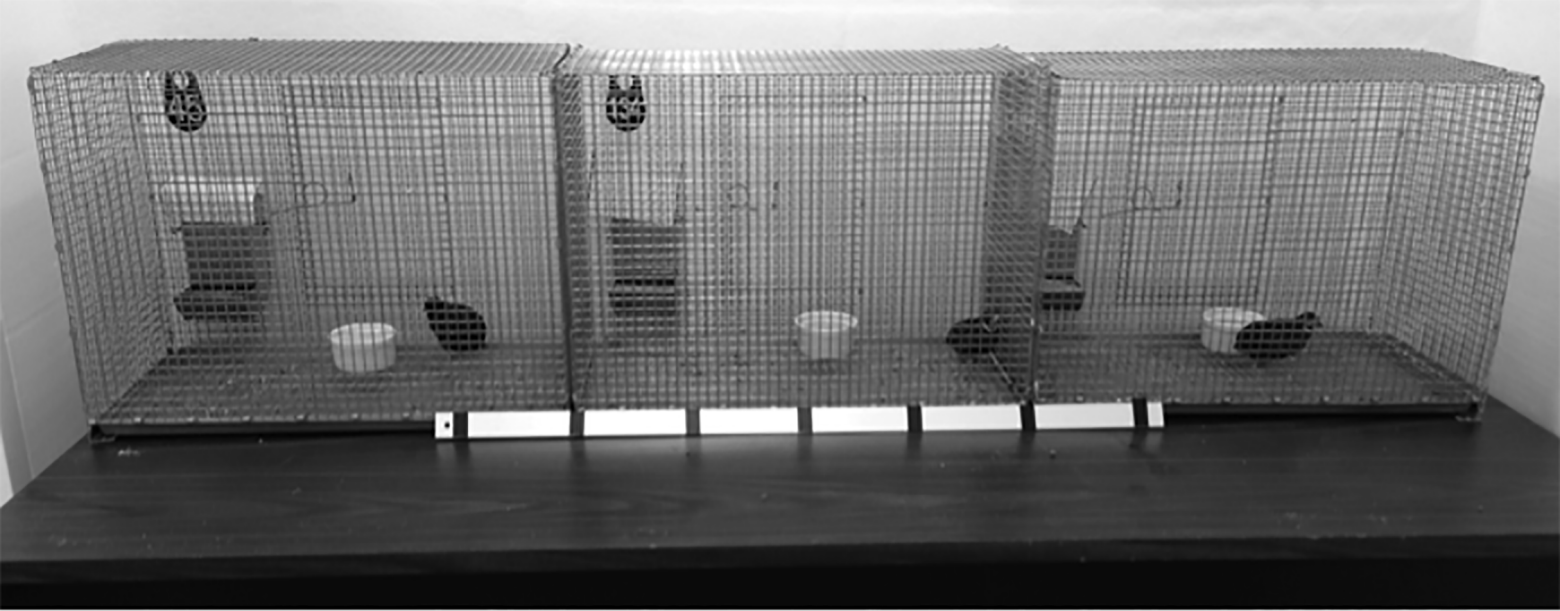
Experiment 1. The cage arrangement for phase 2. The male subject was in the center cage, with the two females (familiar cohabitation partner or neighbor and unfamiliar female) on either side. The black lines on the metal bar delineate the left and right proximity zones (quarters) of the male’s cage.

Recorded in two-choice tests or test phases in which there were two females, one on either side of the male’s cage. The total time in sec that the male’s entire body, including head, was within the zone (quarter) of his cage closest to each female was recorded (see Fig 1).

### Statistics

Because the data distributions deviated markedly from normality, data were analyzed with non-parametric tests (McNemar’s or Wilcoxon tests for within-male comparisons and Fisher’s exact tests or Mann-Whitney *U*-tests for between-male comparisons) using Instat (GraphPad Software, San Diego, CA). All tests were two-tailed.

## Experiment 1: Behavior of Males Cohabiting with a Female Compared with Males with a Female Neighbor

The purposes of this experiment were to: (1) determine whether proximity time in a two-choice procedure predicts subsequent allopreening and huddling with a cohabitation partner, (2) determine whether separation followed by reunion elicits these behaviors, and (3) determine whether cohabitation and potential mating are required in order for males to allopreen and huddle with a female. Two-choice tests have been used to measure the partner preferences of pair-forming mammals such as prairie voles and marmosets (*Callithrix penicillata*) [1,23], and separation-reunion tests have been used to elicit allopreening and clumping in zebra finches [11,13].

### Methods

The birds in this experiment had never been in a cage together with another bird as reproductively mature adults. The subjects were 19 males randomly assigned to two groups. Each male in the cohabitation group (*N* = 11) was introduced into the cage of a randomly assigned female of the same plumage type as the male, one pair per day. For the first seven of these males, the introductions took place in the testing room, and the first hour together was video recorded, after which the cage was moved back into the housing room and the male and female continued to live together. For the other four males, introductions occurred in the housing room and the first hour was not video recorded. All cohabiting pairs were provided with nest material (shredded coconut fiber). Brief (5 min) observations were made one or more times daily prior to testing. Each male in the neighbor group (*N* = 8) had a randomly assigned female of the same plumage type housed immediately adjacent to his cage with no opaque barrier between them, so that the two birds were separated by wire cage material plus a 2 cm gap. This allowed social interaction and familiarity but not body contact or mating. Neighbor group males and female neighbors each had nest material provided. Neighbors were arranged one per day on the same days that cohabitation pairs were formed, so that the two groups were matched for the dates they entered the experiment.

Each male was then tested one week after beginning cohabitation or neighbor housing. Immediately before the test, cohabiting males and females were separated into different cages. Each test consisted of three phases. In phase 1 (15 min), the male subject, the female cohabitation partner or neighbor, and a second female were in the testing room in separate cages with opaque barriers to prevent visual contact. The second female had the same plumage type as the familiar female in five of the 16 tests, had never been adjacent to the male in the housing room, and will be referred to as the unfamiliar female, whereas the cohabitation partner or neighbor will be referred to as the familiar female. Phase 1 served to allow the birds to habituate to the room, as a period of visual separation from the familiar female, and to record male vocalizations during separation. In phase 2 (30 min), the two female cages were moved immediately adjacent to the male’s cage, one to the right and one to the left, with the opaque barriers removed, in order to measure proximity time in addition to other non-contact social behavior (Fig 1). Assignments of the two types of females (familiar vs. unfamiliar) to the right or left sides were reversed for each test. In front of the male’s cage was a metal strip marked with black tape to delineate four equal sized zones. Left and right proximity zones were the quarters closest to the left and right female cages, respectively. In phase 3 (30 min) the cage with the unfamiliar female was removed from the testing room and the male and familiar female were moved into the same cage together. This constituted a resumption of cohabitation for the males in the cohabitation group. For neighbor group males it was the first time they had been in the same cage with their familiar females. Thus the total testing time was 75 min and the three-phase procedure included both a two-choice proximity test and a separation-reunion test.

### Results and Discussion

During the first hour when the first seven cohabitation group males were in a cage together with their assigned females in the testing room, no pairing behavior or mating was seen. Instead, four pairs showed aggression, one male in another pair did multiple wings-down displays, and other birds actively avoided each other and tried to escape. During the daily observations of the cohabiting pairs in the housing room, however, eight of the 11 pairs were seen allopreening and/or huddling, some (six) as early as 24 or 48 h after cohabitation began, including three of those that had showed aggression during the first hour together. Three of the 11 pairs were still highly aggressive, especially the females, or trying to escape after 24 h, and to prevent injury these were separated and removed from the experiment, leaving eight males in the cohabitation group. Thus most but not all male-female pairs progressed rapidly to a relationship marked by allopreening and/or huddling, even if they were initially aggressive and even though the birds did not have a free choice of partners.

In phase 1 a majority of males in both groups emitted a very large number of growls and a few loud calls as well (Table 1). There was no statistically significant difference between the two male groups for either vocalization (growls: *U* = 24, *p* = 0.44; loud calls: *U* = 28.5, *p* = 0.75).

**Table 1:** Loud call and growl vocalizations by males in the three experiments. Entries are the number of males that made the vocalization at least once and the median (range) number of vocalizations.

Experiment	Phase	Group or test female (F)	N		Loud calls			Growls	
				# males	median	range	# males	median	range
1	1	Cohabitation	8	6	4	0–46	6	54	0–93
	1	Neighbor	8	5	5	0–36	8	62	24–106
	2	Cohabitation	8	2	0	0–3	2	0	0–72
	2	Neighbor	8	2	0	0–15	3	0	0–63
	3	Cohabitation	8	3	0	0–15	3	0	0–84
	3	Neighbor	8	2	0	0–8	2	0	0–31
2		Cohabitation F	7	1	0	0–28	1	0	0–21
		Neighbor F	7	4	1	0–7	0	-	-
3			9	0	-	-	1	0	0–20

**Fig 2:**
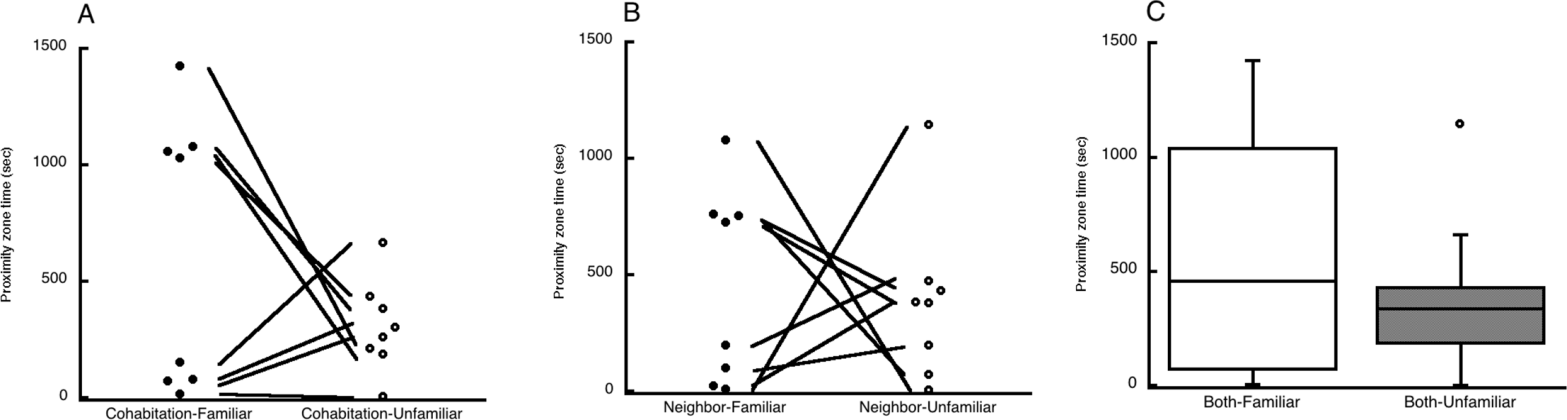
Experiment 1. Proximity zone times in phase 2. A: Time spent by the cohabitation group males in proximity to their cohabitation partners (familiar females) and unfamiliar females. Lines connect the two data points for each male. B: Time spent by the neighbor group males in proximity to their neighbors (familiar females) and unfamiliar females. C: Box plots of the proximity times for the two groups combined.

In phase 2 most males spent a sizable proportion of the test time in the proximity zones, but there was no statistically significant difference within either group or within both groups combined between the time spent with the familiar female (cohabitation partner or neighbor) vs. the unfamiliar female (Fig 2) (cohabitation group: *T* = -9, *p* = 0.25; neighbor group: *T* = -16, *p* = 0.84; both groups combined: *T* = -46, *p* = 0.27). Nor was there any statistically significant difference between the two groups of males in time spent in either proximity zone (time in familiar female zone: *U* = 26.5, *p* = 0.6; time in unfamiliar female zone: *U* = 29.5, *p* = 0.83). In five of the 16 tests (three for the cohabitation group and two for the neighbor group), the male showed aggressive behavior toward the unfamiliar female, lunging at her or doing the wings-down display at her. No male showed any aggression toward the familiar female. Whether the two females had the same plumage type was not related to either proximity time or the occurrence of male aggression. Vocalizations were less frequent than in phase 1, and too few males vocalized at all for a statistical comparison between the groups (Table 1).

**Fig 3:**
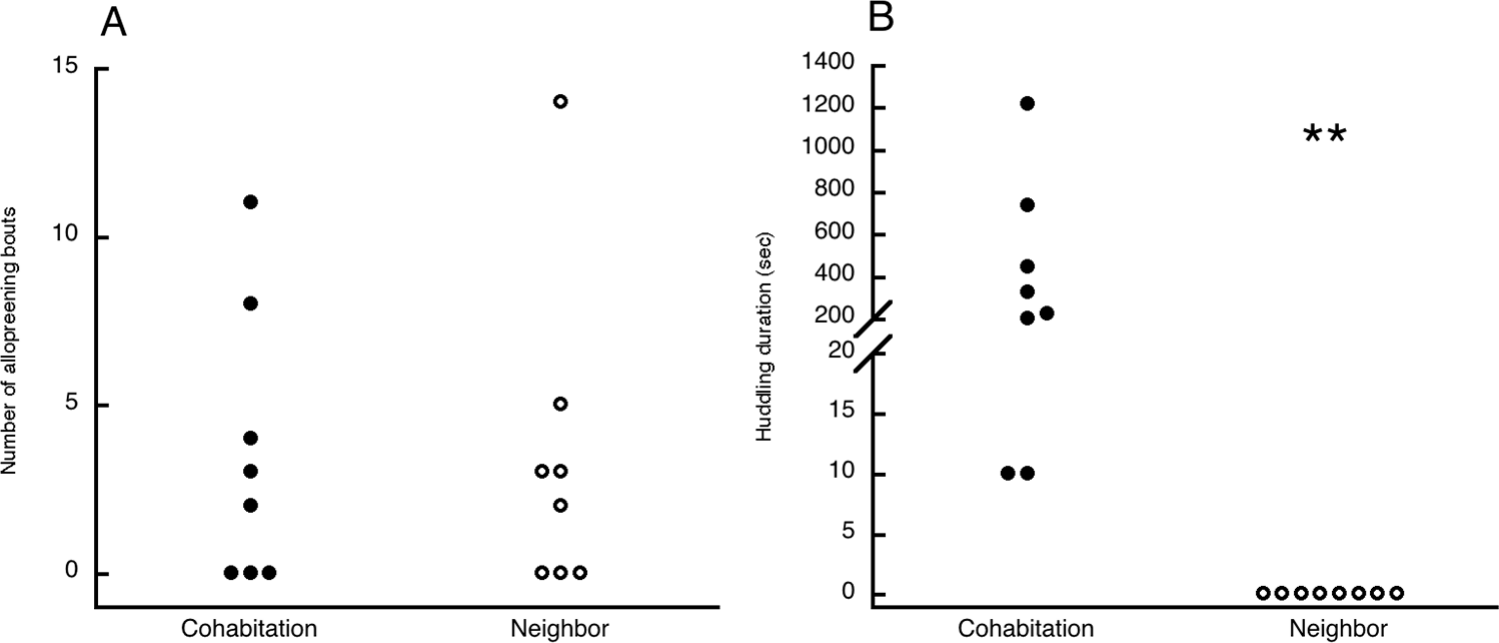
Experiment 1. Pairing behavior in phase 3 during reunion with the cohabitation or neighbor females. A: Number of allopreening bouts initiated by the males in the cohabitation and neighbor groups. B: Time spent huddling with the female by males in the cohabitation and neighbor groups. ** *p* = 0.0002.

In phase 3 a majority of males in both groups allopreened the familiar female, and neither the proportion of males (5/8 in each group) nor the number of allopreening bouts initiated by the males differed by group (Fig 3A) (number of bouts: *U* = 35, *p* = 0.96). All the males in the cohabitation group huddled with the female, whereas none of the neighbor group males did (Fig 3B), a highly statistically significant difference between the groups (*p* = 0.0002). No males in either group showed the wings-down display or any other aggression. Only two males, both in the cohabitation group, attempted to copulate, and one completed the mating. As in phase 2, too few males vocalized at all for statistical comparison between the groups (Table 1).

Time spent near the familiar female in phase 2 predicted whether the male would initiate allopreening bouts with her in phase 3, but mainly for the neighbor group. Males that allopreened had higher familiar female proximity times than males that did not allopreen for the neighbor group (*U* = 0, *p* = 0.036) and for both groups combined (*U* = 8, *p* = 0.016), but not for the cohabitation group alone (*U* = 3, *p* = 0.25).

The results of this experiment allow several conclusions. First, the separation-reunion paradigm is effective for reliably eliciting two very distinctive pairing behaviors: allopreening and huddling. Second, cohabitation is required for the pair relationship to develop to the point where huddling will occur in the separation-reunion test, but familiarity without cohabitation (and without being able to mate) is sufficient for the relationship to develop to the point where allopreening will occur, behavior not seen during the first hour that unfamiliar birds are cohabiting. Third, when birds cohabit a relationship marked by allopreening and huddling can form quickly (in as little as a day) even if it begins with aggressive behavior. Most but not all assigned pairs formed such relationships. Fourth, males presented with familiar females, either initially or as a reunion, seldom attempt to copulate.

In studies with prairie voles and several other socially monogamous species, proximity in two-choice tests has been used as a measure of the formation of a pair relationship, and proximity is referred to as affiliative behavior [1,23]. In this king quail experiment, the results for the proximity test phase were mixed with respect to whether proximity is related to that kind of affiliation (pairing preference or tendency). Proximity times to familiar females were highly variable and bimodal and only predicted subsequent allopreening for the neighbor group. Seven males in the experiment (three in the cohabitation group and four in the neighbor group) spent more time near the unfamiliar female. The occasional occurrence of male aggressive behavior in these tests suggests that proximity can reflect aggression instead of or in addition to pair behavior. The males’ reactions to the sudden appearance of the unfamiliar females may have been similar to the predominantly avoidant or aggressive reactions of the cohabitation group males when first introduced to their assigned and initially unfamiliar females. Also, if paired birds become aggressive toward other birds, then proximity of paired birds might reflect pairing with the familiar female and aggression toward the unfamiliar female in the same test. In zebra finches, behavior in two-choice proximity tests is also an unreliable indicator of pair formation. This is not because of aggression, but instead because the birds normally live in flocks, are strongly social, and are interested in approaching unfamiliar individuals as potential flock mates [24]. Proximity in two-choice tests is a measure of affiliation motivated by copulation preference in experiments with Japanese quail, which, however, do not go on to allopreen or huddle and seldom attack each other [25].

The large number of growls in phase 1 had not been anticipated. It was not clear whether these or the loud calls were occurring because there were females in the room that were not visible, because of separation from the familiar female, or for some other reason. Once the females could be seen (phases 2 and 3), vocalizations occurred much less frequently (Table 1). In order to gain further insight into the context for the male vocalizations in phase 1, each of four males that were not subjects in Experiment 1 and had never cohabited or had a visible female neighbor were moved to the testing room and recorded for 15 min alone in the room. Three of the four emitted large numbers of growls (13, 73 and 36 growls) and two emitted some loud calls (6 and 3 calls). Thus growls occur in large numbers independently of the male’s pairing status or whether there are any other birds in the room.

## Experiment 2: Selectivity of Pairing Behavior

The pairing behavior of socially monogamous species is selective, that is, specific to the opposite-sex pair partner. In prairie voles and zebra finches, non-copulatory physical contact positive social behavior such as allogrooming and huddling or clumping only occurs between pair partners. In prairie voles, proximity in two-choice tests also functions as a measure of the selectivity of male-female contact behavior, because males spend more time with the partner than with a novel female [1]. In Experiment 1, proximity of male king quail did not promise to be a valid way to assess the selectivity of pairing behavior because too often it was accompanied by aggressive behavior. Also in Experiment 1, birds that had been neighbors but without cohabiting did not behave aggressively toward each other when placed in the same cage, and instead allopreened. Experiment 2 capitalized on this second finding by using separation-reunion tests to see if cohabiting males’ allopreening and huddling behavior becomes limited to the cohabitation partner even if the other female is familiar, a strong test of the selectivity required to indicate social monogamy.

### Methods

The subjects were nine males that had not been used in Experiment 1 and had never been in a cage together with another bird as adults. To form cohabiting pairs, each male was first given a female neighbor for one week, that is, a female in an immediately adjacent cage with the opaque barrier removed. This was done in case pairs could then be formed with less initial aggressive and escape behavior. At the end of the week the male and female were moved to the same cage to begin cohabiting and a new female was put into position as the immediately adjacent neighbor. Female cohabitation partners and neighbors had to be assigned without trying to match plumage types. Tests were then carried out one week later. Thus at the time of testing males had been cohabiting with one female for two weeks and were familiar with the neighboring female to the same extent (one week) as for the singly housed neighbor group males in Experiment 1.

To compare the males’ pairing and aggressive behavior toward the two females (cohabitation partner and familiar neighbor) following separation and reunion, each male was tested twice, once on each of two successive days. In one test, the male and his cohabitation female were each placed in the testing room in separate cages separated by an opaque barrier. After 30 min of separation, they were combined into the same cage and video recorded for 30 min. In the other test, the male and his neighbor female were each placed in the testing room in separate cages separated by an opaque barrier. After 30 min, they were combined into the same cage and video recorded for 30 min. The order of the two tests was counterbalanced.

### Results and Discussion

Even when allowed prior familiarity, two assigned cohabitation pairs were too aggressive, especially the females, and were separated and removed from the experiment, leaving seven males as subjects. As in Experiment 1, brief separation followed by reunion was effective for eliciting allopreening and huddling with the cohabitation partner. All seven males showed either allopreening (6/7) or huddling (5/7) or both (4/7) with those females. Their behavior toward the familiar neighbor female was quite different, however. Only one male allopreened or huddled with the neighbor. In that case huddling was initiated by the female and the male then joined in. Thus more males allopreened or huddled with their cohabitation female than with their neighbor female (*p* = 0.031). No males showed any wings-down displays or other aggressive behavior toward their cohabitation females, but a majority of them (5/7) were aggressive toward their neighbor females, repeatedly lunging at and attacking them, or (in one case) threatening and pecking her. One male performed the wings-down display 23 times at his neighbor female, alternating with lunging at and attacking her. Another male’s test was marked by extensive aggression but by the neighbor female. No attempted matings occurred in this experiment. Only one male growled. There were more loud calls in tests with neighbor females (Table 1), but the difference was not statistically significant (McNemar’s test: *p* = 0.38; Wilcoxon test: *T* = -5, *p* = 0.63).

Thus the pairing behavior of most of the males was specific to the cohabitation partner, even though the neighbor female was also familiar. Furthermore, males that had been cohabiting were aggressive toward the familiar neighbor females, whereas the singly housed males in Experiment 1 were never aggressive toward their familiar female neighbors and instead allopreened them. In other words, most males behaved as if a neighbor is a female to pair with if he was not already paired, but a female to attack if he was already paired.

## Experiment 3: Memory for the Cohabitation Partner

This goal of this experiment was to determine whether king quail still recognize a former cohabitation partner after a longer separation. Birds might behave differently toward former partners than new individuals, providing evidence that they still recognize and remember the partner. A number of studies have shown that birds can recognize and remember their mates’ vocalizations, and the pair relationship of zebra finches is maintained during visual separation as long as the birds can still hear each other [26,27]. Male Japanese quail have been shown to recognize individual females, even though they do not display the kind of social behavior with them that occurred in Experiments 1 and 2 and instead simply copulate [28].

### Methods

The subjects were nine males that had cohabited with females in Experiments 1 and 2 but had been separated from them for 9–10 weeks since then. During the separation they were housed singly adjacent to other males, with opaque barriers separating them from neighbors, and housed so they could not see their former cohabitation females. During the separation the males could hear all the vocalizations of all the other birds in the housing room, including their former cohabitation females. Each male was given a single two-choice test. For each test the male subject and two females were each placed in separate cages in the testing room, separated by opaque barriers, with the male in the center cage (as in Fig 1). One female was the male’s former cohabitation partner. For seven of the subjects, the other female was another male subject’s former cohabitation partner. For the other two subjects, the other female had been the familiar neighbor of a male (not a subject in this experiment) in Experiment 1 and had allopreened him. Thus all the females had previously been paired or had engaged in allopreening or huddling, and each male’s two choices differed in whether they had engaged in that behavior with him. The female that had not been with him had never been his adjacent neighbor with or without a separating barrier, and will be referred to as the unfamiliar female. Females were only roughly matched for plumage type (silver with white or wildtype with speckled), because there were not enough females left for closer matching. The female positions were counterbalanced across tests. Once the three birds were in place, the barriers were removed and the test recorded for 30 min.

### Results and Discussion

**Fig 4:**
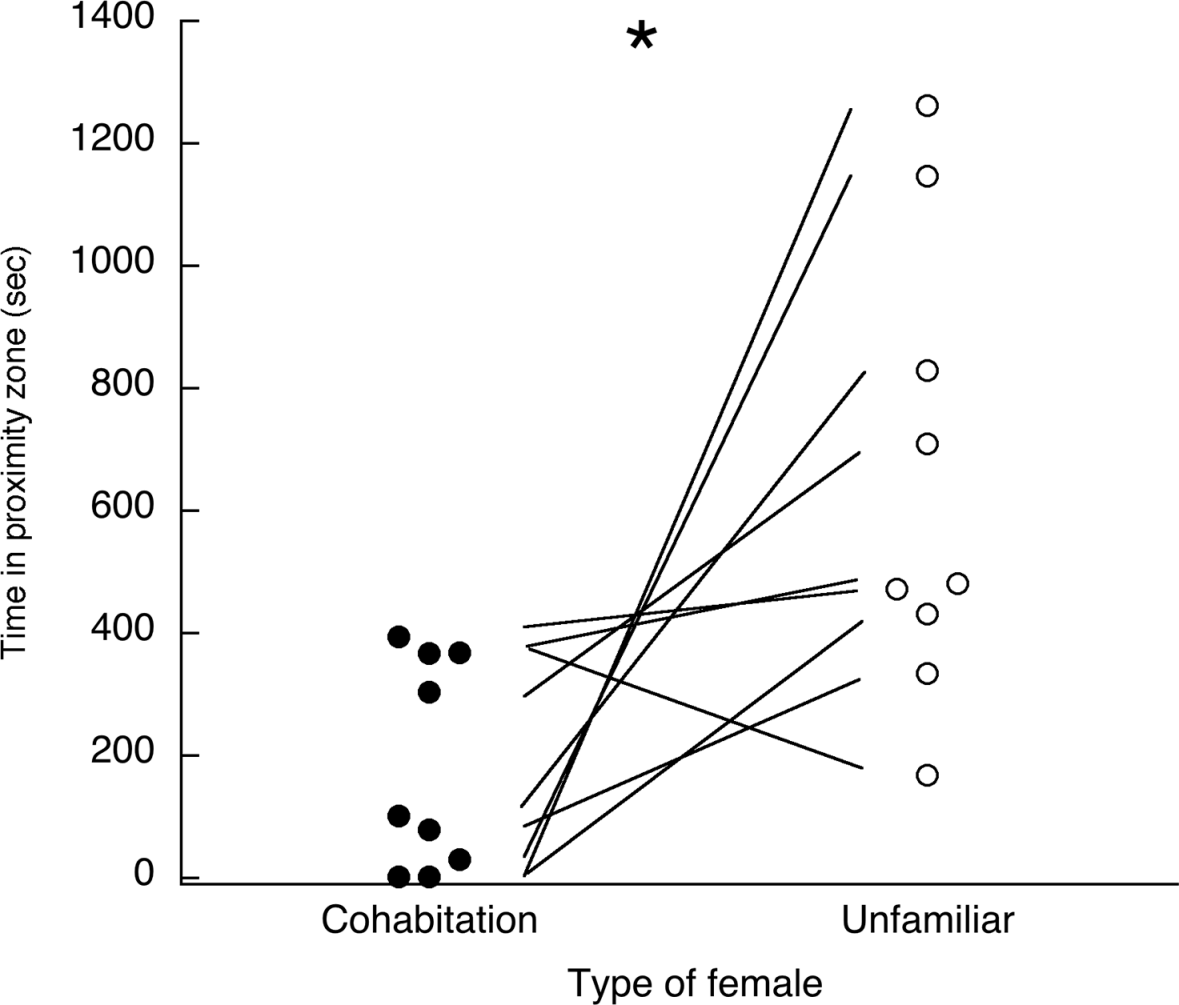
Experiment 3. Shown are times spent by males in the proximity zones near their former cohabitation females vs. the unfamiliar females following a multi-week separation. Lines connect the two data points for each male. * *p* = 0.02.

All but one male spent more time in the proximity zone of the unfamiliar female than in the zone of his former cohabitation partner (Fig 4) (*T* = -3, *p* = 0.02). Three males lunged at or did wings-down displays only at the unfamiliar female and two others showed some kind of aggressive behavior toward both females. Only one male emitted growls and none emitted loud calls (Table 1).

Clearly there is some kind of recognition that is reflected in the males’ proximity times. The males may have recognized and remembered the plumage type of their former partners. Females gave loud calls in only one test, but there were other vocalizations (ticking sounds and chattering) that could have also provided cues for recognition. In light of the results of Experiments 1 and 2, it could have been aggressive motivation that produced the greater time spent near the unfamiliar female. There is also the possibility, however, that the females are responsible for the results in addition to or instead of the males. Although the former cohabitation females approached the males (that is, spent some time very close to his cage) in five of the nine tests, the unfamiliar females always approached the males, and in two tests lunged at them aggressively. There is the possibility that aggressively motivated approaches by the unfamiliar females stimulated the males to spend more time near them in order to engage in an aggressive interaction. Regardless of which bird is driving the results, the males’ behavior ended up being quite different toward the two females.

## General Discussion

The results of these experiments show that king quail readily display the pairing behavior of allopreening and huddling when the sexes are allowed to cohabit and are given short separation-reunion tests. Even with random assignment to partners, most pairs progress rapidly from avoidance or aggression to allopreening and huddling. This behavior is specific to the cohabitation partner. Cohabiting males are aggressive toward other females, even familiar females, whereas unpaired males show pairing behavior toward familiar females. Birds seem to recognize and remember a former cohabitation partner for at least ten weeks following separation. The results of these experiments are consistent with the reports from the field and from zoos that this is a socially monogamous species with close pair bonds [18,19].

Although most birds are socially monogamous, research using experimental manipulations to test hypotheses about underlying hormonal and neural mechanisms for the formation and maintenance of pair relationships has been largely limited to zebra finches [13]. The experiments reported here establish king quail as a highly suitable species for the study of avian pairing behavior. The behavior is distinctive and easily measured. The birds have the same practical advantages as Japanese quail, including ease of hatching, rearing, and housing, short development time, and disease resistance [29].

Prairie voles and marmosets become aggressive toward other conspecifics as they form a pair relationship [1,23], which reflects the development of a jointly defended territory. Zebra finches are not territorial (they only defend the nest itself) and do not become aggressive toward other birds as they pair [30]. Paired prairie voles spend more time near the partner in two-choice proximity tests, whereas paired marmosets spend more time near an unfamiliar conspecific given a similar choice [1,23]. Zebra finch responses in two-choice tests are driven mainly by overall gregariousness [24]. Thus proximity in two-choice tests is a useful measure of socially monogamous partner preference in some species but not others. In Experiment 1 male king quail did not show greater proximity to the cohabitation female and in Experiment 3 they showed greater proximity to the unfamiliar female. The motivation for king quail proximity was not always obvious, but when it occurred together with other behavior, the behavior was aggressive. King quail are not described as territorial, and instead are often sighted or heard in groups [18]. Thus the function of the aggression shown by the birds in the experiments is not known.

The experiments also provided information about some of the other behavior of this little-studied species. Males never did any kind of strutting (including the wings-down display) to a female once they were cohabiting, even following a brief separation, and wings-down displays were often accompanied by lunging and attacking. This suggests a strictly aggressive function consistent with the “wings-down attack” name given by Schleidt et al. [21]. In the king quail tests, only males ever growled, and much more growling occurred when males could not see other birds, consistent with its description as a “separation call” [19]. Male Japanese quail emit a vocalization that is somewhat acoustically similar, but it is associated with strutting [31], whereas king quail growls never occurred together with wings-down displays in the experiments. The loud long-distance advertisement call of male Japanese quail is the crow, which attracts females [32]. Males crow much less when in visual contact with a female [33]. The loud calls of the king quail occurred only occasionally during the tests but were emitted by females as well as males.

The most important outcome of these experiments is the striking contrast between the behavior of king quail and Japanese quail. In the king quail, only two copulation attempts were seen in over 18 hours of video recordings of males and females together in the same cage. Instead, close and often mutual allopreening and huddling characterized male-female interactions once initial aggression or avoidance subsided. Japanese quail, on the other hand, are famous for vigorous short latency copulation whenever males and females are placed in the same cage. They have been important subjects for studying the hormonal and neural mechanisms for avian copulatory behavior and the consequences of copulation for fertilization success [16,29]. Although one recent study with Japanese quail interprets the results of separation-reunion tests as indicating pair bonds [34], allopreening and huddling have never been reported in this species [17]. Proximity in choice tests predicts copulation, and females aggregate to try to avoid copulatory attempts by non-preferred males [25,35].

Male Japanese quail have a large testosterone-regulated foam-producing gland that functions in sperm competition, suggesting sexual selection exerted by females mating with multiple males, whereas male king quail have no obvious foam gland [36, Adkins-Regan, personal observation]. The mating system of wild Japanese quail has not been clearly established, but reports of genetically near-wild birds in large outdoor enclosures and of wild birds of the sibling species, *Coturnix coturnix*, suggest a flexible mating system with short-term serial male-female associations and mate switching [37,38]. Madge and McGowan [18] describe the Japanese quail mating system as highly variable depending on sex ratio and density, and along with *Coturnix coturnix* they seem to be the least reliably monogamous of the old world quail [39]. The resolved phylogenetic relationship between king and Japanese quail is not yet clear, and at various times king quail have been placed in a different genus, previously *Excalfactoria* and most recently *Synoicus* [40]. Nonetheless, all current phylogenies place the two species in the same small clade (tribe Coturnicini) within the larger phasianid clade (pheasants and allies) of the galliform (chicken-like) birds, along with several other small old world species that are reported to be monogamous [18,39,40,41].

Regardless of the limited information about the mating system of either king or Japanese quail in the wild, there is clearly a pronounced contrast between the overt behavior patterns of the sexes together—allopreening and huddling with little copulation in king quail and high frequency copulation with no allopreening or huddling in Japanese quail. Because few birds, including zebra finches, have relatives with a different mating system, the behavioral differences between these two quail species thus offer a new and unique opportunity for a comparative approach to discovering the neuroendocrine and neural basis of pairing behavior in birds. A logical next step would be to test mechanism hypotheses in birds of both species that were raised under the same conditions and tested in the same way. A comparison of the underlying mechanisms in the two quail species, when combined with the important work that has already gone on with voles, will help reveal which mechanisms are likely to generalize across the multiple independent evolutionary origins of monogamy in vertebrates [42]. The two quail species also raise interesting questions about the ultimate causes of the difference in the behavior of the sexes with each other. King quail are smaller and have a tropical distribution. Japanese quail, like the sibling species *Coturnix coturnix*, breed mainly in the temperate zone and their flexible mating system seems to be the derived state in their clade [39]. Whether or how size or climate were involved in the evolution of the species difference are also questions for the future.

## Supporting Information

S1 Video A male (left) and female (right) king quail allopreening and huddling.
(MOV)
